# Report of Discussions of the Third Regular Meeting of the Western Dental Society

**Published:** 1857-06

**Authors:** 


### REPORT OF DISCUSSIONS OF THE THIRD REGULAR MEETING OF THE WESTERN DENTAL SOCIETY.

### FIRST DAY—MAY 20.

On taking the chair, Dr. Forbes, of St. Louis, in a few appropriate remarks, touched upon the importance of social intercourse among the members of the profession—said that our profession is called the twin sister of Medicine—directed attention to the great responsibility resting upon practitioners of our art, considering it greater than that of other professions, not excepting either Medicine or Theology—alluded to the first meeting of the Society, held in this city about a year ago, and called attention to the various methods of practice discussed at that meeting, mentioning the necessity of reviewing our operations for the past year, and of inquiring how far each member had been benefitted by these discussions.

Dr. HALE, Sen., of St. Louis, responds, thanking the Society for the honor conferred upon him by electing him its first President.

AFTERNOON SESSION.—Subject: *What is the best method of arresting decay in teeth?*

Each member requested to describe his treatment of some particular case.

Dr. HALE, Sen., takes cavity in posterior surface of first bicuspid. After cleaning the cavity in a proper manner, and preparing gold in smoothly folded blocks, adapted in size to the size of the cavity, he first places a conical block in that portion of the cavity nearest the gum—then a block is introduced on each side, then one opposite the first, and then fills up the middle—condenses and finishes in the usual way.

Dr. SPALDING, of St. Louis, takes a very simple case, that of a cavity in the grinding surface of molar. He first enlarges the orifice until the walls of the cavity are perpendicular, or nearly so; then slightly rounds the margin of the cavity, just enough to remove the sharp angles of the corners, and fills with cylinders, condensing the first one against the posterior wall, and so on till the cavity is full; then introduces a tapering point near the middle of the filling, condensing in every direction, and completes the plug by the introduction of a conical cylinder.

Dr. ALLPORT, of Chicago, describes his treatment of cavity in approximal surface of superior central incisor. If the adjoining surfaces are parallel, would separate with file, and when possible, remove the entire cavity by filing. When the crowns are conical in shape, separates by pressing apart, and fills the cavity, avoiding the file as a means of separation when the teeth are to come into contact after the operation—uses cylinders in all approximal cavities.

Dr. DUNHAM, of St. Louis, exhibits his mode of filling cavity in anterior approximal surface of inferior lateral incisor—cavity large, extending nearly or quite down to cutting edge. Separates widely with file, fills with fold, using serrated instruments for packing—by their use is enabled to fill rapidly and to succeed in filling very shallow portions of the cavity, especially the point at or near the cutting edge of the tooth—thinks such fillings are not as hard as those composed of cylinders, but believes they are sufficiently dense, and are usually successful—thinks more teeth are lost by the failure of fillings, and a consequent unwillingness on the part of patients to submit to a second operation, than from any other cause.

Dr. M'KELLOPS, of St. Louis, takes the case of a compound cavity in inferior second molar, involving both approximal surfaces, and extending entirely across the grinding surface. Makes a wide V shaped separation between first and second molars, and a narrow parallel one between the tooth involved and that standing behind it—first fills the body of the anterior cavity with cylinders, finishing at the top with annealed foil, used after Arthur's method, and extending the same towards the central cavity—then fills the body of the central cavity also with cylinders, connecting with the anterior by the use of annealed foil, as before—then fills the posterior cavity in a similar manner, allowing the projecting ends of the cylinder to rest against the wisdom tooth, and completes the filling by uniting the posterior and central portions by the use of annealed foil, as before—all projecting portions are afterwards dressed off with a file.

Dr. LEWIS, of Quincy—Concurs with Dr. Allport, in the treatment of cavities in the incisor teeth—removes the cavity by the use of the file, when that can be done—the proper method has been already described by others.

Dr. Blake, of St. Louis—The subject before the Society for discussion is the best method of filling an incisor tooth.

The case I present is a lateral, badly decayed on the front approximal surface, the lower portion having broken away.

On examination, I find the only part of the tooth which can be retained is from the center of the cutting edge to the surface of the tooth where it enters the gum.

As teeth of this description may be preserved and restored to their natural shape, I will briefly give the method I adopt in their treatment.

Before I proceed, however, I wish to remark that I do not in all cases advise filling teeth that are thus decayed. Formerly, all of this class and condition were made to give place to artificial work ; but now, with the improvements in our art, it is otherwise. I would also say that I am indebted to Dr. Arthur, of Philadelphia, and to Dr. Nichols, a member of this association, for important suggestions on this subject.

I will now suppose a patient seated in the chair, with an incisor as above described. After examining it and its neighbors, if I determine on its preservation, I lay all my plans, then proceed with the operation—first, with a file I make the edges even and smooth, against which I wish to build my filling, then with excavators and drills, carefully clean and prepare the cavity. If the nerve is exposed, I treat and remove it, then fill the fang, which leaves a good attachment for the plug ; but if it is possible to preserve the nerve, I in all cases do so. If I do not attach to the filling of the fang, I usually make two places of attachment, one near the upper portion of the cavity, and the other as near the lower as the strength of the tooth will permit. Having thus prepared the cavity, I proceed to anneal the gold.

Have generally used Dunlevy's for this purpose, but recently have been using foil manufactured by our worthy Secretary, Dr. Leslie. I divide a sheet into three equal parts, and fold so as to make my strips about one-fourth of an inch in width. I cut these in pieces about five-eighths of an inch in length. I

now place these pieces, as many as convenient, without touching each other, on a very thin piece of platina, and heat over a spirit lamp, using a blowpipe, so as to raise a white heat.

Having a sufficient quantity prepared and all things ready, I direct the patient to take as easy a position as possible, telling him it will require some time to complete the operation. I proceed to dry the cavity, using prepared cotton.

I always use serrated instruments for these plugs, but seldom have more than three points. I generally take three pieces of gold to commence, but after making the attachment to the tooth, use only one piece at a time, carefully and thoroughly condensing as I proceed.

In this manner, if kept dry, we may build out to any extent we wish. I build to a little more than the full size of the tooth—then dress down with files to its proper size, giving it as natural a shape as possible. I next burnish, then stone down and burnish again. It is now ready for fine pulverized pumice stone, which I use with soft wood, also with tape. I again burnish lightly, which gives a high finish. As this, however, would strike unfavorably the eye of the beholder, I touch it over carefully with pumice. This completes the operation.

Dr. BARRON, of St. Louis, exhibits his treatment of compound cavity in superior central incisor; uniting both approximal surfaces, extending across the cutting edge, and accompanied by the loss of the lower portion of the crown to the extent of one-third of its whole length. First, fills with upright cylinders, extending as far down as the enamel is sound, reserving a small point in one corner, where he commences the use of crystal gold, with this substance he builds down, and thus restores the tooth to its original form and size, gold supplying the place of the lost dentine.

Dr. SILVERS, of St. Louis, takes cavity in grinding surface of molar. Prepares cavity same as Dr. Spalding—fills with rough pellets, thinks them more adhesive than cylinders, and more readily compressed. In condensing, opens clear down to bottom of cavity—thinks a wedge or cone, placed in the middle, would be liable to be driven out by an antagonizing tooth, when it strikes the side of the plug—has never used cylinders, and thinks he never shall.

Dr. STURGISS, of Quincy, takes a case of a dishing cavity located in one cusp of grinding surface of molar. Cuts small grooves in sides of cavity, and fills with foil in rolls, sometimes uses the fold.

Dr. PEEBLES, of St. Louis, heard from Dr. Hale last year about filling fangs with plaster of Paris—tried this substance in fangs of lower molars in the case of a poor patient—in one tooth found it difficult to introduce, in the other, was introduced easily—filled crowns with gold, successful so far.

Dr. PERRINE, of St. Louis, gives a case of use of plaster in filling fang of lower bicuspid. After five months, tooth became troublesome—on removing gold filling, found the plaster soft, unfavorable result.

Dr. P. also describes case of large cavity in superior cuspid, involving death of nerve and the loss of posterior portion of crown—lower cuspid, owing to loss of back teeth, striking into the cavity—filled fang with foil, then completes the operation by building up against the thin front wall of enamel, after Arthur's plan of using annealed gold. Feels more confident of success in the use of foil in this manner than in the use of crystal gold.

Dr. HUBBARD, of Quincy, takes a central cavity in grinding surface of molar, with four diverging lines of decay. First opens central cavity with drill, then enlarges the diverging lines, fills first the lateral lines, then the posterior, then the central cavity, finishing with the anterior radiating line.

Thinks it not necessary to remove every portion of decayed matter from the bottom of the cavity, especially if in so doing, the nerve would be uncovered—thinks the formation of new osseous matter will occur in young persons, in the course of six or eight weeks. Sometimes uses gutta percha over exposed nerves—believes we destroy too many nerves, and often do so when it is not necessary. Does not succeed in more than one-half the cases in which he has practised filling fangs.

Dr. PORTER, of Michigan, exhibits his treatment of cavity in approximal surface of superior incisor, accompanied by loss of corner on cutting edge—fills partly with foil, and partly with crystal gold—rolls foil into rope, then unrolls and rolls again, to produce increased roughness—uses instruments with serrated points, allows the foil in packing to rest against the adjoining tooth, builds down with crystal gold, to restore the broken corner to original shape.

Dr. LEVETT, of St. Louis, takes a case similar to the last. Uses the fold or strip, and builds out with foil, annealed and used in the manner recommended by Dr. Arthur, of Philadelphia.

Dr. BULL, of Lasalle, treats sensitive dentine with solution of gutta percha—after a few days removes gutta percha, and fills with foil twisted into a rope.

Dr. TAFT, of the Ohio Dental College, remarked, When a case is presented, he takes into consideration the constitutional tendencies and temperament of the patient—also, the strength of the vital forces, the liability to irritation, and the probable influence of remedial treatment.

This diagnosis is made up from external indications, also by these forms an opinion as to the character of the teeth. These indications are found in the development and condition of the various tissues, the color and tone of the skin, etc.; thus we can know the general character of the teeth before a special examination is made. We can not operate alike on all teeth. Some would be destroyed by the same operation that would save others, owing to constitutional differences. These differences are too frequent ly disregarded, even by those whom we consider good operators.

We will take, for description, a cavity of the anterior surface of superior molar. The decay occupies almost the entire spproximal surface. The first step is the special examination, to ascertain the nature and extent of the decay, the form of the cavity, and the strength of the walls. Then separate. There are two methods of accomplishing this—by pressure, and by cutting. The latter only is applicable in this class of cases. He uses the chisel instead of the file in cutting—can cut more rapidly by this means, and with less annoyance to the patient. Smoothes the cut surface with a fine file.

The next step is to remove all the decay. Though in some cases, where the nerve would be exposed, it may be admissible to permit a small portion of partially decomposed bone to remain for a covering, but in no case should we leave even the slightest portion of decaying bone about the orifice of the cavity, or even upon the walls. Then give the cavity such form as will certainly retain the filling. It is very seldom the case that when the decay is removed, the cavity is of the proper shape; and hence, the formation of the cavity constitutes another step.

The cervical wall of the cavity should be a little inclined inwards, the lateral and crown walls, at about a right angle.

For drying the cavity, uses prepared cotton, sometimes blotting paper; after this, throw in a jet of warm air, drying the cavity to whiteness.

In filling either with crystal gold or foil in blocks, commence the filling at the cervical wall of the cavity, filling down two-thirds to the crown wall, consolidating perfectly to the lateral walls. Then fill to the crown wall of the cavity, filling up to the portion first introduced, terminating the introduction of the gold somewhere near the center of the filling.

In the application of force for the consolidation of fillings, it should be a general rule to apply the pressure as nearly on a direct line with the axis of the tooth as possible. Consolidate the surface perfectly, and finish with the file, pumice, Scotch stone, and buff or burnish.

In these decays, the crown wall is often broken away, leaving the two lateral walls standing, with strong projections or angles. There are two methods of operating in such cases. First, to cut down these angles back as far as the crown wall is broken away. That makes the surface at the lateral borders diagonal with the axis of the tooth, and the surface of the filling will partake of the same inclination. And second, the angles, if firm, may be left standing, and the filling introduced and brought out flush with the angles; thus forming the filling with an approximal and a crown surface. The angle formed by the filling should not be too sharp.

On the subject of building out fillings, Dr. ALLPORT said, that from some things that had been said, he feared there was some misapprehension as to his views on this point. He did not practice it generally, but would resort to it only occasionally; believes that more was being done in that way just at this time than would be done a few years hence. If practitioners were not careful, they would ride this hobby too hard.

Dr. SPALDING said he did not like the practice, excepting in very rare cases—would hardly resort to it at all in back teeth, and very rarely in front ones.

THURSDAY MORNING.—Subject: *The causes and treatment of diseased gums.*

Dr. SPALDING said he would pass over, for the present, the first part of the question, relating to causes, and confine himself to that portion relating to treatment only. In the first place, he would, of course, carefully and thoroughly remove all accumulations of foreign matter, repeating the operation as often as might be necessary, at the same time also excising any excessive prurient growth of the gums. The next step would be the employment of such means as would serve to assist nature in restoring a healthful condition to the diseased parts. He had long ago laid aside the use of astringents in cases of this kind, and relied altogether upon the use of stimulants. In the local treatment demanded in these cases, it had been his object to rouse up and stimulate the action of the secretory and absorbent vessels, and he had met with better success than formerly, when employing astringents. He relied chiefly upon the different alkalies, and would be guided in his selection mainly by the taste, choosing those which were found the least unpleasant. Directed the patient to make free use of the brush, using as firm a one as the gums would bear—considered the friction of the brush highly useful in effecting a cure.

Dr. ALLPORT does not rely much on medicines. Carefully removes all accumulation, scarifies freely, and rubs with a soft brush to promote depletion—subsequently directs the use of Prof. Westcott's recipe, consisting of equal parts nitrate of potassa and carbonate of soda, dissolved in a strong decoction of sage, sweetened to improve the taste, to be used after each meal.

Dr. DUNHAM : Sometimes finds it necessary to remove teeth as well as tartar. Employs scarification and also excision. Sometimes uses nitrate of silver, but always avoids a resort to such active remedies, if possible. As a wash, relies mainly on tinc. myrrh, made with rectified whisky instead of alcohol; also uses carbonate of soda and nitrate of potassa—knows of nothing as good as simple tinc. myrrh, which is astringent, stimulant and antiseptic.

Dr. HALE, Sen., said, the principal causes were neglect, want of cleanliness, and consequent accumulation of tartar. After the necessary cleaning, treats with astringent washes and powders, such as myrrh, tannin, etc. Where suppuration of the gums exists, finds the case difficult to treat with success; regards periostitis as the primary disease in these last cases, and the suppuration of the gums as secondary; thinks friction and cleanliness are much more important than the use of astringents in all cases, regarding the employment of friction as indispensable to a cure.

Dr. BLAKE considers tartar accumulations as a leading cause—carefully removes, then scarifies, and also uses artificial leeches as an additional means of depletion—uses tinc.

myrrh, with the addition of a little creosote—thinks astringents valuable.

Dr. PHILLIPS, of Mo.—Cleanses. Scarifies freely, to produce depletion, and sometimes employs astringents, not often.

Dr. STURGISS—Thinks diseases of the gums hereditary—irregularity also a cause ; uses myrrh, but does not generally find its use followed by a permanent cure.

Dr. SILVERS mentioned case in which he employed mechanical support to retain the teeth in their places—treated with nut galls, sulph. ether, and creosote.

Dr. LEVETT, after cleaning and scarifying, uses borax and honey, also astringents—thinks diseases of the gums hereditary, especially where there is a venereal taint.

Dr. PEEBLES—Is an inquirer. Has not yet satisfied himself in regard to his own treatment; has used many remedies recommended by others, but has not found them effectual. Has found long "gourd-seed" teeth more subject to diseases of the gums and alveoli. Can not cure such cases short of extraction.

Dr. PERRINE—Has nothing new or additional to offer; has a case of necrosis of alveoli. Has failed with astringents, and now proposes to adopt the use of stimulants.

Dr. HUBBARD—Nothing to add, the ground having been already gone over—relies very much upon depletion by scarification—has used astringents and alkalies—has met with success in the use of solution of nitrate of silver—meets with cases that he can not manage—thinks nitrate of silver has a direct action on the mucous membranes.

Dr. PORTER—Nothing to add ; uses in extreme cases nitrate of silver, in ordinary cases, recommends use of soap, also uses astringents, and scarifies.

Dr. BRANCH, of Galena, uses both astringents and stimulants. If the teeth are of the long gourd-seed variety, thinks the leverage greater in mastication, such teeth having also openings between them near the gum. Food there accumulates, and by mechanical pressure on the gums, produces absorption and disease. Reports a case of removal of tumor from the gum by ligature, producing a cure. In ordinary cases, depends chiefly on friction from the use of the brush; directs the employment of a hard brush, to produce bleeding; objects to excision, preferring to wait the operation of nature, and in cases of suppuration of the margin of the gum, advises extraction of the teeth; for acute periostitis, prescribes spirits of nitre, saturated with calcined alum.

Dr. TAFT remarked, There are cases that do not yield to any ordinary treatment. Such cases are usually influenced by both local and constitutional causes. When the latter exists, constitutional treatment is indicated, but when it is the result solely of local causes, then local treatment only is indicated. This consists of excision occasionally, depletion, and counter-irritation by scarifying. The medicinal treatment consists of astringents, stimulants and tonics. When the vital tone is low, slight local causes will produce disease. In some instances, the most heroic treatment is indicated—in others, touch lightly. Constitutional treatment may require the aid of a physician. He referred to a case. A lady, 22 years of age, good constitution. Found considerable constitutional disturbance and lassitude; gums much enlarged, covering roots and some sound crowns. The free margin of the gums presented a rounded mass of one-half to three-fourths of an inch in diameter. The patient used compound ext. of colocynth. Excised the enlarged portion with the bistoury, passing it entirely around the arch, at about the line of the gum margin in health. Removed all the roots and some of the teeth, and on the second day afterward, removes any of the excrescences that have been overlooked; used astringent wash two or three days, then used nitrate of silver in substance. The mouth entirely restored in five or six weeks. Prefers excision to waiting the slow process of absorption.

Dr. FORBES says there are three principal causes, local, constitutional and scrofulous. Cleanses thoroughly—is not particular to avoid laceration. Excises with pointed scissors in some cases. Prescribes calcined alum and pulv. cinnamon. Uses creosote, either pure or diluted, in periostitis; also uses calcined alum and creosote; uses tinct. of myrrh in all cases.

Dr. HALE, Sen.. treats common cases with powdered nut gall or tannin, followed by nitrate of silver in solution—generally successful in cases of children.

AFTERNOON SESSION.—(Miscellaneous discussion not reported.)

FRIDAY MORNING.

*Discussion on Report of Committee on Patents, &c.*—Dr. Spalding objects to the phraseology of the report.

Dr. Allport is opposed to patenting any thing beyond instruments and other articles of manufacture. If instruments or materials on which a patent has been obtained are thrown into the market and freely sold to any and all who desire to purchase, he has no objection to a patent—but he is strenuously opposed to the sale of office rights in any case whatever.

Dr. Branch offers a substitute changing the phraseology of the pending resolution.

The whole report is recommitted, and the committee retire.

Report on Cheoplasty by Dr. Taft, chairman, presented and adopted without debate.

Resolutions on patents are again reported and discussion resumed.

Dr. Spalding was not prepared to oppose the *entire* principle of patenting, as seemed to be contemplated in the first resolutions reported by the committee—did not feel willing to go to that extreme. The government had instituted the patent system for the purpose of protecting inventors in their rights. Men who had expended their time and money in perfecting improvements in the mechanic and other arts, could only in this way secure to themselves the results of their own labors and expenditures. He considered copy-righting as only a part of the same general system, extending protection to authors as well as inventors, and if instruments and other articles of manufacture could be sold in the unrestricted way in which books were, he had no objections to a patent. He was willing that inventors should exercise the same control over the manufacture of patented articles that authors did over the publication of copy-righted books, and that both inventors and authors should look wholly to the sale of their productions for remuneration for their labors. He agreed with the views held by Dr. Allport; indeed, Dr. A. had exactly expressed his mind on this subject. Neither did he approve the course taken by some, of shielding themselves under the name of others. If it was right for a professional man to hold any interest in, or control over a patent, it was not only right, but in his opinion far more honorable for him to take a frank, open and straight-forward course and hold it in his own name. But when any man belonging to a liberal profession claimed the right of patenting any mode of practice, and proposed, directly or indirectly, to peddle out office or territorial rights, he, for one, would go as far as any body towards heaping upon such a custom all the censure which it merits at our hands. He had drawn a distinction in this matter of patents which he conceived to be the correct one, and he was glad that so many whose opinions he valued were of the same way of thinking with himself.

Dr. Barron said that the second resolution offered by the committee qualified the first, and as chairman of that committee, he was willing to so modify the first as to meet the views of members. For himself, he was strongly opposed to the growing custom of resorting to patents, and if some check was not put upon it, it would soon be the case that a Dentist could not go into his office and commence any operation without first buying a patent instrument, and then purchasing the right to use it.

Dr. Branch is not opposed to the custom of patenting. Thinks if it is right to patent at all, it is right in one profession as well as another; thinks we should fix upon some rule for the guide of patentees or of inventors, and should say that the principle is either right or wrong.

Dr. Allport thinks the resolution before us as it now stands only condemns the course pursued by some, and does not cover the whole principle of patenting; thinks that no member of a liberal profession has a right to first patent an instrument or material and then patent its use. He alluded to the very excellent dental chair invented and patented by his friend, Dr. Perkins. When a man bought one of Dr. P.'s chairs, the right of use went with the chair; and, as in the case of a book, it might be sold and resold, and still the last owner had just as much right to its use as the first. The example of Dr. Perkins was worthy of all imitation. He had not only constructed the best chair ever yet made, but he had placed it within the reach of every Dentist, so that all might enjoy the benefit resulting from its use.

FRIDAY—3 P. M.

*Difficult Dentition.*—DR. SPALDING spoke of the importance of the subject under consideration; thought that a large per cent. of the mortality among infants could be fairly referred to this cause; thought physicians have not given the subject that careful attention to which it was entitled, owing, no doubt, to the wide range of subjects which necessarily come within the scope of medical practice. Said the subject fairly came within the province of the dentist, and earnestly hoped that the members here present would take up the matter in good earnest, was himself comparatively ignorant, but would like to hear from Dr. Taft, who has long studied the subject, and could, he believed, impart much important information.

DR. TAFT said there are various causes of difficult dentitions. In some cases there seems to be a want of assimilating powers; in other cases an imperfect supply of the proper material. This is manifested by a deficiency in the development of the osseous structure, and sometimes extends to the muscular and other organic tissues also. When the deficiency is general, it falls within the province of the physician. This deficiency extends to the teeth, and affects them as well as other bony structures. Usually there is a deficiency of bone phosphate. To supply this, the administration of phosphate of lime has been resorted to, and with very marked success.

It may be given to the child or to the mother while the child is nursing, or to both; this influences the eruption of the teeth, by causing a more rapid development. No difficulty arises from its exhibition; it is readily appropriated, but in different degrees. Have gone a little farther back than this. A mother had three children, and in each case their primary teeth decayed very early; indeed, they seemed to be scarcely through the gums till they began to decay.

The mother used bone phosphate for four months previous to the birth of the child, and while the child was nursing. That child had firm, well organized teeth, that seem as though they will resist decay till the irruption of the permanent ones.

Related a case of the rapid restoration of a child 16 months old, that was reduced to an extreme degree of emaciation and debility, The cause seemed to be difficult dentition. General treatment was continued much as before, and the administration of bone phosphate adopted at once. The child immediately began to improve, and in two months seemed to be entirely restored, cutting six teeth soon after beginning the use of the phosphate; gave child from two to six grs. per day in two portions; when administered to the mother, from 6 to 15 grs. per day, in two portions.

Its use is not indicated when the fontanelle closes readily, and there is no apparent want of bone material.

